# The Impact of the COVID-19 Pandemic on Cancer Mortality in Pennsylvania: A Retrospective Study with Geospatial Analysis

**DOI:** 10.3390/cancers15194788

**Published:** 2023-09-29

**Authors:** Savanna G. Ledford, Fritz Kessler, Jennifer L. Moss, Ming Wang, Eugene J. Lengerich

**Affiliations:** 1 Department of Public Health Sciences, College of Medicine, The Pennsylvania State University, Hershey, PA 17033, USA; sledford18@gmail.com (S.G.L.); jlm837@psu.edu (J.L.M.); 2 Penn State Cancer Institute, Hershey, PA 17033, USA; 3 Department of Geography, College of Earth and Mineral Sciences, University Park, The Pennsylvania State University, State College, PA 16801, USA; fck2@psu.edu; 4 Department of Family and Community Medicine, College of Medicine, The Pennsylvania State University, Hershey, PA 17033, USA; 5 Department of Population and Quantitative Health Sciences, Case Western Reserve University, Cleveland, OH 44106, USA; mxw827@case.edu

**Keywords:** COVID-19, cancer mortality, geospatial analysis, Pennsylvania

## Abstract

**Simple Summary:**

The COVID-19 pandemic may have impacted cancer mortality rates by disrupting patient health behaviors and access to cancer care, as well as being directly affected by infection with SARS-CoV-2. There is limited population-based research that investigates the impact of the COVID-19 pandemic on cancer mortality in the United States. The aim of our retrospective study was to quantify the change in cancer mortality from before the COVID-19 pandemic to during the COVID-19 pandemic in Pennsylvania. We found that mortality rates did not continue their previous downward trajectory and twenty-six counties had an increase in the rate in 2020. The 2015–2019 rates were positively associated with the 2020 rates, and the impact of sociodemographic and geographic factors on the 2020 rates varied by county. Our findings suggest a negative effect of the pandemic on cancer mortality in Pennsylvania and had a varied effect on its counties. Future research could confirm our findings, and if confirmed, may inform cancer research, care, and outreach.

**Abstract:**

Background. We sought to quantify the impact of the COVID-19 pandemic on cancer mortality and identify associated factors in Pennsylvania. Methods. The retrospective study analyzed cross-sectional cancer mortality data from CDC WONDER for 2015 through 2020 for Pennsylvania and its 67 counties. The spatial distributions of 2019, 2020, and percentage change in age-adjusted mortality rates by county were analyzed via choropleth maps and spatial autocorrelation. A Wilcoxon Signed Rank Test was used to analyze whether the rates differed between 2019 and 2020. Quasi-Poisson and geographically weighted regression at the county level were used to assess the association between the 2019 rates, sex (percent female), race (percent non-White), ethnicity (percent Hispanic/Latino), rural–urban continuum codes, and socioeconomic status with the 2020 rates. Results. At the state level, the rate in 2020 did not reflect the declining annual trend (−2.7 per 100,000) in the rate since 2015. Twenty-six counties had an increase in the rate in 2020. Of the factors examined, the 2019 rates were positively associated with the 2020 rates, and the impact of sociodemographic and geographic factors on the 2020 rates varied by county. Conclusions. In Pennsylvania, the 2020 cancer mortality rates did not decline as much as reported before the COVID-19 pandemic. The top five cancer types by rate were the same type for 2019 and 2020. Future cancer control efforts may need to address the impact of the COVID-19 pandemic on trends and geospatial distribution in cancer mortality.

## 1. Introduction

The COVID-19 pandemic may have impacted cancer mortality by disrupting preventive health behaviors and access to cancer treatment. However, quantitative research on the impact of the COVID-19 pandemic on cancer mortality is limited. In the United Kingdom, the COVID-19 pandemic may contribute to approximately 3500 additional deaths from cancer of the breast, colon/rectum, lung, and esophagus due to the suspension of cancer screening, deferral of diagnostic work, and the discontinuation of interventions for non-urgent, non-symptomatic patients [1]. In the United States, there was an increase in colorectal, pancreatic, breast, and prostate cancer mortality during the COVID-19 pandemic due to the postponement of interventions such as surgery and onsite cancer care [2]. A separate study during the COVID-19 pandemic concluded that a five- to six-month surgical delay for patients with stage two renal masses had worse overall survival compared to those who had only a three- to four-month surgical delay [3]. 

Examination of the impact of the COVID-19 pandemic on the association of geographic and sociodemographic factors on cancer mortality is also warranted because cancer mortality has not been equal across space for all groups. For example, in Georgia, counties that had a greater percentage of non-Hispanic Black individuals were older, had increased poverty, and more rural counties had elevated breast, colorectal, lung, and prostate cancer mortality rates before the COVID-19 pandemic [4]. In addition, rural counties with the highest gastrointestinal cancer mortality rate were in the southeastern region of the United States [5]. 

Pennsylvania, the location of this study, also has important geographic and sociodemographic differences in cancer mortality. Cancer is the second leading cause of death in Pennsylvania [6]. The rate of cancer mortality in Pennsylvania during 2020 was 153.2 per 100,000, compared to the national rate of 144.1 per 100,000 [7]. Compared to females, males had a higher mortality rate for colorectal cancer, lung cancer, leukemia, and melanoma in 2016 [6]. African Americans had a higher mortality rate from lung or bronchus cancer (49.0 per 100,000) compared to the White population (40.2 per 100,000) in 2016. Black women had a higher breast cancer mortality rate (27.3 per 100,000) compared to White women (20.8 per 100,000) in 2016 [6]. There was also geographic variation in cancer mortality in Pennsylvania. For example, the counties of Philadelphia (53.6 per 100,000), Clinton (54.2 per 100,000), Fayette (54.8 per 100,000), and Forest (62.1 per 100,000) had the highest lung cancer mortality rates from 2012 to 2016, compared to the state rate of 40.9 per 100,000 [6]. 

The impact of the COVID-19 pandemic on cancer mortality, at the state and county level, in Pennsylvania, as well as the county-level correlates of mortality change, have not been previously reported. Knowledge of the changes in cancer mortality during the COVID-19 pandemic will add to our understanding of its impact. In addition, these changes may inform future cancer control efforts. Therefore, the objectives of this study were to: (1) quantify the change in the cancer mortality rates at the state and county level, (2) analyze the presence of clustering of cancer mortality rates at the county level, and (3) identify factors, including sociodemographic and geographic factors, associated with cancer mortality during the COVID-19 pandemic. 

## 2. Materials and Methods

### 2.1. Study Design and Data

The study used a retrospective design with cross-sectional data for the years 2015 through 2020 to gain a broad view of the mortality trends. Our primary analysis sought to examine the change in cancer mortality between 2019 (pre-pandemic) and 2020 (during the pandemic). The outcome variables were age-adjusted mortality rates at the state and county level, in which cancer (ICD-10, C00-C97) was the underlying cause of death [7]. Age-adjustment was the direct method used and the United States 2000 standard population age distribution was used as the standard. 

For explanatory variables in the county-level analysis, we included sex (percent female), race (percent non-White), and ethnicity (percent Hispanic/Latino) from the U.S. Census Bureau for 2020 [8]. We also used socioeconomic status (SES) and rurality (rural–urban continuum codes, RUCC) data from the Agency for Toxic Substances and Disease Registry and United States Department of Agriculture Economic Research Service, respectively [9,10]. SES was a county-level value that was the sum of the percentage of individuals below the 150% poverty estimate, the percentage who were at least 16 years of age and unemployed estimate, the percentage of housing cost burden of occupied housing units estimate, the percentage without a high school degree estimate, and the percentage who were uninsured estimate for each county based on 2020 U.S. Census data with a range of values of 0.55 to 4.77 were used. RUCC codes for each county were dichotomized into metro/urban (RUCC 1-3) and non-metro/rural (RUCC 4-9).

### 2.2. Statistical Analysis

We first examined the annual trend from 2015 to 2020 in the cancer mortality rates for Pennsylvania and calculated the annual percentage change between years. T-tests were used to identify whether mortality data between population groups were statistically significant. At the county level, we used a Wilcoxon Signed Rank test (for paired comparisons) to determine if the 2019 rates were different from the 2020 rates. We also visualized the rates at the county level with choropleth maps. We tested for spatial autocorrelation with Local Indicators of Spatial Association (LISA) maps and calculated Moran’s I value, and related pseudo *p*-values. To examine the association of 2019 rates as well as sociodemographic and geographic factors with the 2020 rates, we used quasi-Poisson regression. Quasi-Poisson regression was used due to the overdispersion of the 2020 rates. We developed three quasi-Poisson regression models to quantify the association of factors with the 2020 rates: model 1: 2019 rates; model 2: 2019 rates and sociodemographic factors; model 3: 2019 rates, sociodemographic factors, and RUCC (geographic factor). Geographically weighted regression (GWR) allowed us to quantify the spatial impact of county location on the relationship between the 2019 rates as well as the sociodemographic and geographic factors with the 2020 rates. For GWR, the three models were the 2019 rates (model 1), RUCC (model 2, binary), and model 3 was 2019 rates, sex (percent female), race (percent non-White), ethnicity (percent Hispanic/Latino), and SES. Three counties (Sullivan, Forest, and Cameron) did not report a reliable 2020 rate due to the number of deaths being less than 20. To examine the impact of missing county rates on the quasi-Poisson regression and GWR results, we conducted a sensitivity analysis using the median value of the surrounding counties for any counties that had missing data. 

All analyses were performed using R Statistical Software (v4.1.0; R Core Team 2021), QGIS (3.18.3-Zurich), and GeoDa (1.20.012). This study was determined by Penn State IRB (STUDY00019984) to be exempt from regulatory review because the data were publicly available and de-identified. 

## 3. Results

From 2015 through 2020, Pennsylvania had a declining trend in the age-adjusted cancer mortality rate (Table 1). The rate before the COVID-19 pandemic (2015–2019) decreased from 167.2 per 100,000 in 2015 to 153.5 per 100,000 in 2019, resulting in a total decrease of 13.7 and an average annual decrease of 2.7. However, the rate in 2020 was 153.2 per 100,000, only 0.3 per 100,000 less than the rate in 2019. For each year, the rate was higher among Black/African American versus White populations, non-Hispanic/Latino versus Hispanic/Latino populations, and males versus females (*p*-value < 0.001). White and non-Hispanic/Latino populations and females had a decreasing trend in rates from 2015 through 2020. The rate for males consistently decreased from 2015 through 2019 but then increased in 2020. The change in the rate for other groups was inconsistent across the time period. Black/African American populations had a higher rate (180.2) compared to White populations (152.6) in 2020. Non-Hispanic/Latino populations had a higher rate (154.5) compared to Hispanic/Latino (108.5) populations in 2020. Also, males had a higher rate (181.9) compared to females (132.4) in 2020. 

For Pennsylvania, we observed a negative percentage change in the rate each year from 2015 through 2020 (Table 2). The percentage change for the rate between 2019 and 2020 was −0.2, whereas the annual percentage change for the preceding years ranged from −2.7 to −1.5. White populations, non-Hispanic/Latino populations, and females had a negative percentage change between 2019 and 2020. There was inconsistency in the annual percentage change for other individual groups. For example, Asian/Pacific Islander populations had the largest average percentage decrease in the rate for 2015 through 2019 (−12.6) but had the largest percentage increase between 2019 and 2020 (8.3). Black/African American populations and males had a positive percentage change in the cancer mortality rate between 2019 and 2020 and a negative average percentage change for the preceding years. 

### 3.1. Map Visualization

**Figure 1:**
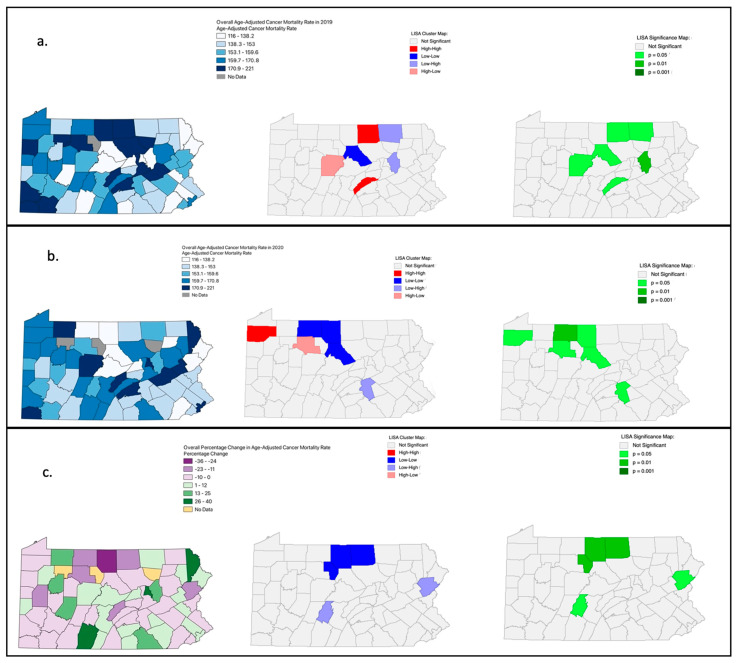
Geographic distribution of the county level age-adjusted cancer mortality rates (per 100,000), LISA cluster, and LISA significance maps for 2019 (**a**), 2020 (**b**), and the percentage change (**c**) in Pennsylvania. COVID-19 and Cancer Mortality in Pennsylvania. (Underlying cause of death, Source: CDC WONDER). Maps were created in QGIS and Geoda.

In 2019 (Figure 1a), one county (Forest; gray shade) did not report a reliable rate. Counties in the north central and southwestern regions of Pennsylvania exhibited higher 2019 rates, compared to the central, northeast, and southeast regions of the state. The LISA significance map (Figure 1a—right map) indicates which counties were contributing significantly to spatial autocorrelation. Columbia County (darkest green shade in Figure 1a map) had high statistical significance (*p* = 0.01) in contributing to the negative spatial autocorrelation (lighter blue shade center panel of Figure 1a). The counties shaded in a lighter green were also statistically contributing to spatial autocorrelation but at a lower *p*-value (*p* = 0.05). The counties shaded in grey were not statistically contributing to spatial autocorrelation. Despite select counties exhibiting local positive or negative spatial autocorrelation, the resulting Moran’s I value and related *p*-value for 2019 (0.005 and *p*-value = 0.27) indicated that the data did not support the global presence of spatial autocorrelation. 

For 2020 (Figure 1b), three counties (Forest, Cameron, and Sullivan Counties, shaded in grey) did not report reliable rates. Unlike 2019, the counties in the north central region of Pennsylvania did not have noticeably elevated rates; however, counties with higher rates were scattered across the state. The LISA cluster map (Figure 1b—center panel) indicated both positive and negative spatial autocorrelation. The LISA significance map indicated that McKean County contributed significantly to the positive spatial autocorrelation (*p* = 0.01), whereas counties in a lighter shade of green contributed significantly to spatial autocorrelation but at a lower *p*-value (*p* = 0.05). Despite the maps suggesting local positive and negative spatial autocorrelation, the Moran’s I value and related *p*-value for 2020 (0.007 and *p*-value = 0.22) did not support global spatial autocorrelation. 

When comparing Figure 1a to Figure 1b, counties that experienced a high rate in 2019 were not necessarily the same counties that experienced a high rate in 2020. However, the Wilcoxon Signed Rank test resulted in a *p*-value of 0.78, indicating that the ranking of mortality rates for the two time periods (2019 and 2020) were not significantly different. For the percentage change in the rate from 2019 to 2020, of the 64 counties that reported a 2020 rate (Figure 1c), 26 counties had an increase in the rate compared to 2019, 1 county (Luzerne) had no change in mortality rate, and 37 counties had a decrease in the 2020. Wayne County (northeast corner) had the largest percentage increase (39.6) in the rate between 2019 and 2020 and Potter County (north central) had the largest percentage decrease (−35.8) in the rate between 2019 and 2020. The LISA cluster map indicated both positive and negative spatial autocorrelation (Figure 1c—center panel). The LISA significance map confirmed the statistical contribution that three counties in the north central region made toward positive spatial autocorrelation (*p* = 0.01). Monroe County and Blair County statistically contributed to negative spatial autocorrelation with a lower *p*-value (*p* = 0.05). Despite the maps suggesting positive and negative spatial autocorrelation, the resulting Moran’s I value and related *p*-value for 2020 (−0.01 and *p*-value = 0.83) indicated that the data did not support global spatial autocorrelation.

### 3.2. Quasi-Poisson regression

In all three models, the 2019 rates were positively associated with the 2020 rates (estimate = 0.002, *p*-value ≤ 0.01) (Supplementary Table S1). With an estimate of 0.002, a 1-unit increase in the county-level 2019 rate results in an estimated increase in the county-level 2020 rate by a factor of 0.002 per 100,000. The regression did not identify statistically significant associations between sociodemographic and geographic factors with the 2020 rates, controlling for the 2019 rates. The results from the sensitivity analysis were not substantially different. 

### 3.3. Geographically Weighted Regression 

The output for each model, i.e., the coefficient range, allowed us to quantify the spatial impact of each factor on the 2020 rate (Supplementary Table S2). For model 1, the 2019 rate coefficient ranged from 0.19 (1 unit change in the 2019 rate resulted in an increase in the 2020 rate by 0.19 per 100,000) to 0.51 (1 unit change in the 2019 rate resulted in an increase in the 2020 rate by 0.51 per 100,000). For model 2, the RUCC coefficient ranged from −3.47 (a change from metro/urban to non-metro/rural resulted in a decrease in the 2020 rate by 3.47 per 100,000) to 4.90 (a change from metro/urban to non-metro/rural resulted in an increase in the 2020 rate by 4.90 per 100,000). For model 3, the 2019 rate coefficient ranged from 0.16 to 0.45. The coefficient for sex (percent of county that is female) ranged from −1.26 to 1.12. The race (percent non-White individuals) coefficient ranged from −0.76 to −0.13. The ethnicity (percent Hispanic/Latino individuals) coefficient ranged from −0.47 to 0.92. Lastly, the SES coefficient ranged from −5.66 to 5.91. The in-sample prediction accuracy for model 1 was 26% with an Akaike information criterion (AICc) value of 526.26. For model 2, the in-sample prediction accuracy was 11% with an AICc of 539.13. For model 3, the in-sample prediction accuracy was 40% with an AICc value of 535.83. Based on the AICc values, model 1 was a better-fit model for the 2020 rates; however, model 3 had a higher in-sample prediction accuracy. However, the sensitivity analysis yielded different results for the range of coefficient values, AICc, and in-sample prediction accuracy. The use of the median value for the counties that did not provide a reliable rate resulted in a decrease in the in-sample prediction accuracy of all three models, compared to the primary analysis. However, both the primary and sensitivity analyses revealed the same overall conclusion in that model 1 was the better fit model based on AICc and that model 3 had the highest in-sample prediction accuracy. 

**Figure 2:**
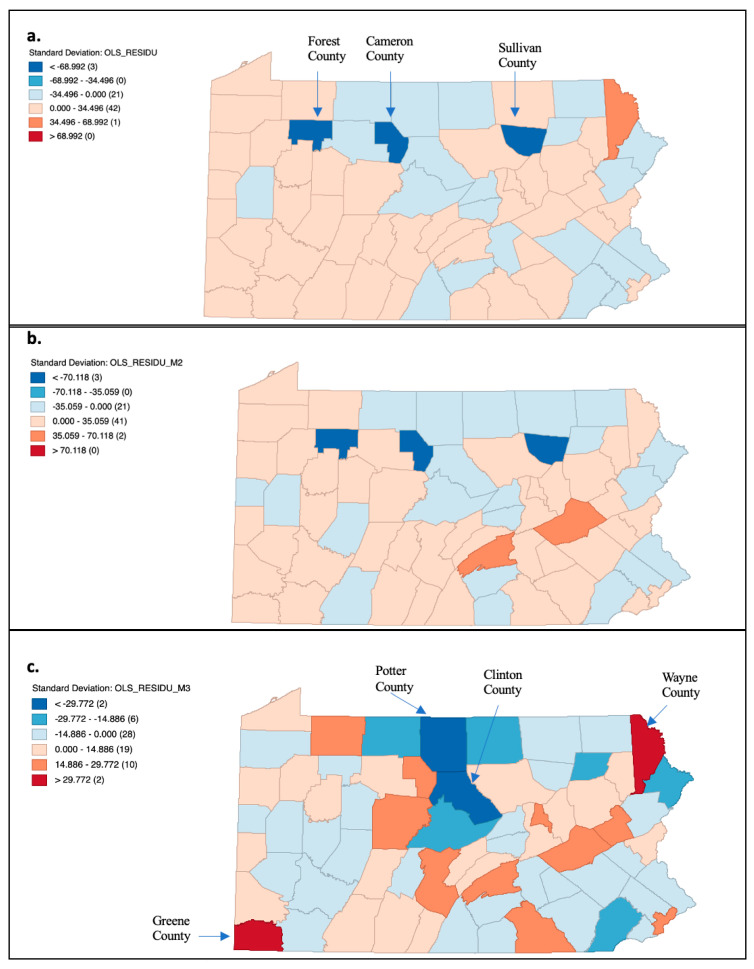
Maps of residual values obtained for the models (model 1 (**a**), model 2 (**b**), and model 3 (**c**)) used for geographically weighted regression for Pennsylvania. COVID-19 and cancer mortality in Pennsylvania. (Underlying cause of death, Source: CDC WONDER). All maps were created in Geoda.

The distribution of the estimated residuals across Pennsylvania counties obtained from the GWR is shown in the maps in Figure 2. The influence of the factors contained in each model on the 2020 rate varied by county. For model 1, the best fit model based on the AICc value, showed that it overpredicted the 2020 rate for three counties (dark blue color). Model 2 also overpredicted the same three counties (Figure 2b). These three counties did not report a reliable 2020 rate value; thus, the model overpredicted the 2020 rate since there was no observed rate for the counties (Figure 2a,b). Model 1 and model 2 did not underpredict county-level 2020 rates. However, in Figure 2c (model 3, highest in-sample prediction accuracy), both under- and over- predictions for the 2020 rates were present. The model overpredicted Potter County and Clinton County (darkest blue) and underpredicted Wayne County and Greene County (darkest red). Therefore, for both Potter County and Clinton County, the observed 2020 rate was not as high as the predicted value generated by model 3. For Wayne County and Greene County, the observed 2020 mortality rate was higher than the rate predicted by model 3 (Figure 2). The sensitivity analysis showed that model 1 underpredicted Wayne County (located in the northeast corner of Pennsylvania) and overpredicted Potter County located in northcentral Pennsylvania). For model 2, the model underpredicted Perry County (located in the central region of Pennsylvania) and overpredicted Clinton County (located in the northcentral region). Model 3 did not underpredict Greene County and did not overpredict Clinton County. 

## 4. Discussion

This study found that there were important and substantial changes in cancer mortality at the state and county level in Pennsylvania from before the COVID-19 pandemic to during the COVID-19 pandemic. From 2015 through 2019, the rate in Pennsylvania demonstrated an average decline of 2.7 per 100,000 each year. However, the state-level rate did not continue to decline at the previous average rate of −2.7 average annual change. For example, if the change in the cancer mortality rate had continued declining at the 2015–2019 average percentage, then the rate in 2020 would be approximately 150.8 per 100,000 instead of the observed 153.2 per 100,000. A declining death rate has been observed across the United States from 2001 to 2020 [11]. 

For comparison, the 2020 age-adjusted cancer mortality rate for the other 49 states had a range from 119.5 to 177.3 with a mean of 147.0 and a median of 146.6. The 2020 age-adjusted cancer mortality rate of 153.2 for Pennsylvania fell within the third quartile of all states. States with the highest age-adjusted rate include Oklahoma (171.1), Mississippi (176.0), Kentucky (177.3), and West Virginia (177.0). In 2019, the age-adjusted rate for the 49 states ranged from 117.2 to 179.1 with a mean of 149.1 and a median of 149.0. Pennsylvania had an age-adjusted rate of 153.5, which fell in the third quartile compared to the other states. As with 2020, the states that had the highest age-adjusted rate were Oklahoma (173.0), Mississippi (179.1), Kentucky (176.4), and West Virginia (175.0).

However, the leading sites of cancer mortality, for both females and males, did not change from 2019 to 2020. In 2020, the top five age-adjusted cancer mortality rates for females were trachea, bronchus, and lung (28.3), breast (19.6), lymphoid, hematopoietic, and related tissue (11.7), colon, rectum, and anus (10.9), and pancreas (10.2). However, for males, it was trachea, bronchus, and lung (41.1), lymphoid, hematopoietic, and related tissue (19.2), prostate (18.0), colon, rectum, and anus (16.3), and pancreas (13.8). In 2019, the top five age-adjusted cancer mortality rates for females were trachea, bronchus, and lung (30.1), breast (19.5), lymphoid, hematopoietic, and related tissue (11.7), colon, rectum, and anus (11.6), and pancreas (10.3). For males, it was trachea, bronchus, and lung (43.2), lymphoid, hematopoietic, and related tissue (19.7), prostate (17.6), colon, rectum, and anus (15.3), and pancreas (13.4). The magnitude of change by cancer type may be an area of future research interest.

Our findings indicate that select populations in Pennsylvania experienced an increase in the cancer mortality rate from 2019 to 2020. These populations were males, Black/African Americas, Asian/Pacific Islander, and Hispanic/Latino populations. Females, non-Hispanic/Latino, and White populations experienced a decrease in the rate in 2020 compared to 2019. Our findings are supported by previous publications indicating that Black/African American populations and males tend to have significantly higher cancer mortality rates [6,11,12]. However, across all populations analyzed, Asian/Pacific Islander populations had the largest percentage increase in the rate in 2020 compared to 2019, but the 2020 rate is not as high as other populations. This is consistent with a previous publication indicating that Asian/Pacific Islander populations had the lowest rate in comparison to other populations [13]. Despite the cancer mortality rate being the lowest among Asian/Pacific Islander populations compared to other populations, cancer is the leading cause of death among Asian Americans [14]. Our results suggest that the COVID-19 pandemic may have exacerbated cancer mortality for Asian/Pacific Islander populations, a population for whom cancer was already the leading cause of death before the COVID-19 pandemic. A 2014 publication found that the increase in mortality among American Indians/Alaska Natives may be linked to a lack of access to medical care [15]. In response, nonprofit organizations, cancer centers, and tribal healthcare facilities have implemented solutions to improve cancer care access, but the COVID-19 pandemic may have limited access to these efforts. 

Additionally, we examined geographic variation across the state. At the local level, we observed scattered instances of counties exhibiting statistically significant positive and negative spatial autocorrelation. While we did not find statistically significant spatial autocorrelation at the global level, we observed a change in the geographic distribution of cancer mortality rates. For example, in 2019, the counties in the north central region had higher rates (counties that were included in the group with the highest rate) but in 2020, counties in that same region were not among those with the highest rates. The north central region tends to be comprised of more rural counties and rurality is a factor that has been identified in previous publications as being associated with increased cancer mortality in a non-pandemic environment [4,5,6].

Both quasi-Poisson regression and GWR found that the 2020 rate was strongly associated with the 2019 rate, but no associations with sociodemographic and geographic factors were identified. We did not observe that high poverty, unemployment, and non-Hispanic Black populations were associated with the change in cancer mortality rates in 2020, as has been reported in other studies [4,5,16]. However, we did see that the impact of the 2019 rates as well as the sociodemographic and geographic factors with the 2020 rates differed by county in Pennsylvania. The impact of 2019 rates, sex, race, ethnicity, rurality, and SES varied by county and was highlighted in the maps of the residual values. Based on AICc values, model 1 (2019 rates) was a better-fit model for the 2020 rates with the model performing well in most Pennsylvania counties (Figure 2a); however, in-sample prediction accuracy was highest for model 3 (2019 rates and sociodemographic factors) but the model did not perform as well due to over- and under-predictions being present (Figure 2c). Future investigations of the relationship of alternative sociodemographic and geographic factors with 2020 rates may improve model performance. In addition, the Wilcoxon Signed Rank test indicated that the rates in the two time periods were not significantly different. A potential explanation as to why the 2019 and 2020 rates were not significantly different may relate to the small unit increases for 2019 and its resulting increase for 2020 identified in model 1 of the quasi-Poisson regression. The quasi-Poisson regression also indicated that only the 2019 rates were significantly associated with the 2020 rates. Specifically, higher 2019 rates may be associated with higher 2020 rates. The addition of the sociodemographic and geographic factors in the models did not change the estimated value of the 2019 rates; thus, the value was not influenced by the additional factors.

Our data also provide insight for specific counties. For example, Wayne County, located in the northeast corner of Pennsylvania, had the highest percentage increase in cancer mortality rate from 2019 to 2020 (39.6%). Wayne County had a RUCC of 6, which a is nonmetro region with an urban population between 2500 and 19,999 adjacent to a metro area [17]. The county also had a greater proportion of people who were 65 and over (24.5% compared 18.6% for the state), fewer hospital beds per capita, higher percentage of the population with no health insurance, and fewer primary care doctors per 1000 capita [17,18]. In addition, 6% of the county population are non-White and had a low socioeconomic status score of 0.59, a medium to high level of vulnerability [17,18]. Therefore, our selected sociodemographic and geographic factors may not be sufficient to explain an increase in the cancer mortality rate during the COVID-19 pandemic. Future research could specifically investigate the health behaviors, hospital cancer care management, and sites and stage of cancer for residents of Wayne County or similar settings.

Additional research could also investigate the association of rurality on cancer mortality at the county level in a pandemic versus a non-pandemic environment. A study in the United States concluded that from 2006 to 2015, the yearly age-adjusted cancer mortality rates for all cancer sites decreased at a slower pace (−1.0%) in the nonmetropolitan areas versus metropolitan areas (−1.6%) [19]. Interestingly, the nonmetropolitan rural areas had a higher incidence and deaths of cancers related to tobacco use as well as screenable cancers [19]. The rural–urban divide has prompted institutions to implement strategies to close the cancer gap [20]. These strategies included the expansion of services, mitigation of financial burden associated with limited insurance coverage, improvement of access to clinical trials, and creation of partnerships to address cancer care gaps [20]. However, these strategies were pre-pandemic and may need to be re-evaluated in a post-COVID-19 era.

Disruption of medical care during the COVID-19 pandemic was demonstrated in the United States and internationally [21]. In Greece, hospital stay and operation duration increased during the COVID-19 pandemic [21]. Also, patients delayed hospital care due to both their anxiety and fear of coronavirus infection [21]. Delayed presentation to a Malaysian medical facility during the pandemic was found to have detrimental effects on clinical outcomes, especially if surgery is required [22]. A study in the United States in 2020 reported a 74% decrease in patient evaluation and management visits among seniors who had cancer [23]. In addition, the pandemic also affected cancer screening [23]. In India, there was a 12% to 50% decrease in cancer surgeries during the COVID-19 pandemic [24]. A separate study, in England, concluded that a three-month delay in surgery for patients with stage one to three cancer was estimated to cause at least 4700 deaths and 92,214 life-years loss per year [25]. Thus, delay in cancer care impacted patient survival. 

In March 2020, healthcare resources were relocated to manage COVID-19 infections. For example, cancer screening programs were suspended in several countries [26]. In the United States, there was a decrease in cancer screening for breast, cervical, and colorectal cancer as more than 50% of centers stopped cancer screening [27]. A decrease of 41% for breast, cervical, colorectal, and lung cancer screening was observed in Canada [27]. In 2020, 9.4 million cancer screening tests did not occur due to patient fear of COVID-19 in a medical setting, staff shortages, or the closure of screening centers [28]. These missed cancer screenings suggests that the COVID-19 pandemic may indirectly cause an increase in the number of future cancer deaths because of the negative relationship between a delay in screening and cancer being diagnosed at an advanced stage [29]. From a United States medical center perspective, screenings for breast, colon, and cervix decreased by 86% to 94% from 20 January 2020 to 21 April 2020 [30]. Also in the United States, breast and cervical cancer screening decreased from 2018 to 2020 by 6% and 11%, respectively; however, no change was observed in colorectal cancer screening [31]. 

Our study had limitations. First, three counties did not report reliable values due to the number of deaths being less than 20 for 2020. Second, the SES variable, comprised of five different measures, provided only an overall assessment of SES rather than understanding the association of a specific SES measure on the 2020 rates. Third, we were unable to create a single GWR model that incorporated all factors of interest. Fourth, we examined only deaths that had cancer as the underlying cause of death. Thus, we did not assess the role that COVID-19 had in a cancer death, or the role of cancer in a COVID-19 death, leading to an underestimated impact of the COVID-19 pandemic on mortality rates. Fifth, we used mortality data from 2020 as an estimate for the experience during the pandemic, when the pandemic was not declared in Pennsylvania until March 2020. However, this limitation would lead to bias that decreased the observed differences between 2019 and 2020. Despite these limitations, the study had at least three strengths. To our knowledge, this was the first study to quantify the change in cancer mortality during the COVID-19 pandemic for an individual state. Second, our study assessed the impact of sociodemographic and geographic factors changes. Third, our study used both spatial and non-spatial analyses for a more thorough understanding of the changes in cancer mortality.

## 5. Conclusions

In Pennsylvania, the COVID-19 pandemic impacted cancer mortality rates at both the state and county level. At the state level, the secular decreases in the annual cancer mortality rate were interrupted. At the county level, the spatial distribution of the cancer mortality rates during the COVID-19 pandemic was different from the distribution before the COVID-19 pandemic. The changes in the 2020 rates were primarily associated with the 2019 rates. Future research could investigate the impact of the COVID-19 pandemic on cancer mortality beyond 2020, the association of other sociodemographic and geographic factors with the cancer mortality during the COVID-19 pandemic, and the impact of the pandemic on the reduction in cancer screening and prevention. This research would contribute to a comprehensive view of the impact of the COVID-19 pandemic on cancer mortality. These results may inform future cancer control efforts in Pennsylvania by quantifying changes in cancer mortality from prior to the pandemic to after the pandemic. Our findings may also inform the methodology of future studies that examine the impact of the COVID-19 pandemic on cancer mortality rates in other states. 

## Figures and Tables

**Table 1:** Annual age-adjusted cancer mortality rate per 100,000 in Pennsylvania, 2015 through 2019 (before the COVID-19 pandemic)) and 2020 (during the COVID-19 pandemic), by race, ethnicity, and sex. COVID-19 and Cancer Mortality in Pennsylvania. (Underlying cause of death, Source: CDC WONDER).

Characteristic	2015 Age-Adjusted Rate	2016 Age-Adjusted Rate	2017 Age-Adjusted Rate	2018 Age-Adjusted Rate	2019 Age-Adjusted Rate	2020 Age-Adjusted Rate
Total	167.2	164.7	161.0	156.6	153.5	153.2
Race						
White	166.5	163.2	159.7	156.2	153.3	152.6
Black/African American	197.9	198.4	195.3	183.6	177.0	180.2
Asian/Pacific Islander	93.2	102.4	90.9	79.4	81.5	88.3
American Indian/Alaska Native	Unreliable	51.0	56.1	Unreliable	Unreliable	42.6
Ethnicity						
Hispanic/Latino	98.4	108.1	107.7	112.7	102.2	108.5
Non-Hispanic/Latino	167.7	165.1	161.9	157.7	155.0	154.5
Sex						
Male	201.4	197.3	191.7	186.8	181.4	181.9
Female	143.1	141.8	139.4	135.5	133.3	132.4

**Table 2:** Annual percentage change for the age-adjusted cancer mortality rate per 100,000 in Pennsylvania for 2015–2019 (before the COVID-19 pandemic)), 2020 (during the COVID-19 pandemic) and the percentage change by race, ethnicity, and sex. COVID-19 and Cancer Mortality in Pennsylvania. (Underlying cause of death, Source: CDC WONDER).

Characteristic	2015–2016	2016–2017	2017–2018	2018–2019	2019–2020	Average Percentage Change for 2015–2019
Total	−1.5	−2.2	−2.7	−2.0	−0.2	−8.2
Race						
White	−2.0	−2.1	−2.2	−1.9	−0.5	−7.9
Black/African American	0.3	−1.6	−6.0	−3.6	1.8	−10.6
Asian/Pacific Islander	9.9	−11.2	−12.7	2.6	8.3	−12.6
American Indian/Alaska Native	N/A	10.0	N/A	N/A	N/A	N/A
Ethnicity						
Hispanic/Latino	9.9	−0.4	4.6	−9.3	6.2	3.9
Non- Hispanic/Latino	−1.6	−1.9	−2.6	−1.7	−0.3	−7.6
Sex						
Male	−2.0	−2.8	−2.6	−2.9	0.3	−9.9
Female	−0.9	−1.7	−2.8	−1.6	−0.7	−6.8

N/A relates to the “unreliable” since a percentage change could not be calculated.

## Data Availability

The datasets analyzed for the current study are available from CDC WONDER, https://wonder.cdc.gov/ accessed on 1 December 2022, U.S. Census Bureau, https://www.census.gov/quickfacts/PA accessed on 16 January 2023, ATSDR, https://www.atsdr.cdc.gov/placeandhealth/svi/interactive_map.html, and USDA, https://www.ers.usda.gov/data-products/rural-urban-continuum-codes.aspx accessed on 16 January 2023.

## Supplementary Materials

The following supporting information can be downloaded at: https://www.mdpi.com/article/10.3390/cancers15194788/s1. Table S1: Quasi-Poisson regression output of the three models to identify the association of county-level factors (2019 rate, sociodemographic, and rurality) with the county-level 2020 age- adjusted rates (per 100,000) in Pennsylvania. COVID-19 and Cancer Mortality in Pennsylvania. (Underlying cause of death, Source: CDC WONDER). Table S2: Geographically weighted regression output of three models to identify the impact of county location on the relationship between county-level 2019 rate, sociodemographic factors, and rurality with the county-level 2020 (per 100,000) in Pennsylvania. The regression applies for the 64 counties that provided a 2020 age-adjusted cancer mortality rate. COVID-19 and Cancer Mortality in PA. (Underlying cause of death, Source: CDC WONDER).
