# Supplementary material for: The Impact of the COVID-19 Pandemic on Cancer Mortality in Pennsylvania: A Retrospective Study with Geospatial Analysis
Source: Cancers (Basel). 2023 Sep 29;15(19):4788. doi: 10.3390/cancers15194788 (PMC10571537; doi:10.3390/cancers15194788)
Supplement: Supplementary file 1 [file cancers-15-04788-s001.zip › Geo_ms_Supp_Table_S1.pdf]

Supplementary Table S1*: Quasi-Poisson regression output of the three models to identify the association of county-level factors (2019 rate, sociodemographic, and rurality) with the county-level 2020 age- adjusted rates (per 100,000) in Pennsylvania. COVID-19 and Cancer Mortality in Pennsylvania. (Underlying cause of death, Source: CDC WONDER).

		**Model 1**		
**Variable**	**Estimate**	**Std. Error**	**95% CI**	**p-value**
Intercept	4.71	0.09	(4.52, 4.89)	<0.001
2019 Age-Adjusted Cancer Mortality Rate	0.002	0.0006	(0.001, 0.003)	<0.001
		**Model 2**		
Intercept	4.82	0.48	(3.88, 5.75)	<0.001
2019 Age-Adjusted Cancer Mortality Rate	0.002	0.0006	(0.0008, 0.003)	<0.001
Sex (Percent Female)	-0.002	0.01	(-0.02, 0.02)	0.85
Race (Percent Non-White)	-0.0009	0.002	(-0.004, 0.002)	0.59
Ethnicity (Percent Hispanic or Latino)	-0.0007	-0.003	(-0.007, 0.005)	0.81
SES	0.005	0.015	(-0.02, 0.03)	0.73
		**Model 3**		
Intercept	4.86	0.51	(3.85, 5.85)	<0.001
2019 Age-Adjusted Cancer Mortality Rate	0.002	0.0006	(0.0008, 0.003)	<0.001
Sex (Percent Female)	-0.002	0.01	(-0.02, 0.02)	0.81
Race (Percent Non-White)	-0.001	0.002	(-0.005, 0.003)	0.57
Ethnicity (Percent Hispanic or Latino)	-0.0009	0.003	(-0.007, 0.006)	0.78
SES	0.006	0.02	(-0.03, 0.04)	0.70
RUCC (0, metro/urban; 1, non-metro/rural)	-0.006	0.03	(-0.07, 0.06)	0.84
