# Supplementary material for: The Impact of the COVID-19 Pandemic on Cancer Mortality in Pennsylvania: A Retrospective Study with Geospatial Analysis
Source: Cancers (Basel). 2023 Sep 29;15(19):4788. doi: 10.3390/cancers15194788 (PMC10571537; doi:10.3390/cancers15194788)
Supplement: Supplementary file 1 [file cancers-15-04788-s001.zip › Geo_ms_Supp_Table_S2.pdf]

Supplementary Table S2: Geographically weighted regression output of three models to identify the impact of county location on the relationship between county-level 2019 rate, sociodemographic factors, and rurality with the county-level 2020 (per 100,000) in Pennsylvania. The regression applies for the 64 counties that provided a 2020 age-adjusted cancer mortality rate. COVID-19 and Cancer Mortality in PA. (Underlying cause of death, Source: CDC WONDER).

		**Model 1**	
**Factor**	**Minimum**	**Median**	**Maximum**
Intercept	76.65	101.11	127.54
2019 Age-Adjusted Cancer Mortality Rate	0.19	0.35	0.51
AICc		526.26	
Global R2		0.26	
		**Model 2 (Binary Factor)**	
Intercept	151.64	154.84	157.52
RUCC (0, metro/urban; 1, non-metro/rural)	-3.47	2.40	4.90
AICc		539.13	
Global R2		0.11	
		**Model 3 (Non-binary Factors)**	
Intercept	60.37	111.20	156.46
2019 Age-Adjusted Cancer Mortality Rate	0.16	0.33	0.45
Sex (Percent Female)	-1.26	0.033	1.12
Race (Percent Non-White)	-0.76	-0.33	-0.13
Ethnicity (Percent Hispanic/Latino)	-0.47	0.047	0.92
SES	-5.66	-0.5	5.91
AICc		535.83	
Global R2		0.40	
